# Immediate Changes in the Elasticity of Tissue and the Pain Pressure Threshold in Cesarean Scar Tissue After a Vacuum Intervention: An Open Clinical Trial

**DOI:** 10.3390/biomedicines13030557

**Published:** 2025-02-21

**Authors:** Ana González-Muñoz, Santiago Navarro-Ledesma

**Affiliations:** 1 Clinical Medicine and Public Health PhD Program, Faculty of Health Sciences, University of Granada, Av. de la Ilustración, 60, 18071 Granada, Spain; 2 Clínica Actium, Avenida Hernán Núñez de Toledo 6, 29018 Málaga, Spain; 3 Department of Physiotherapy, Faculty of Health Sciences, Campus of Melilla, University of Granada, Querol Street 5, 52004 Melilla, Spain; snl@ugr.es

**Keywords:** cesarean section, algometry, scar tissue, pain pressure thresholds, Adheremeter, vacuum therapy

## Abstract

**Background/Objectives**: Cesarean section (C-section) scars are commonly linked to reduced tissue elasticity and increased pain due to adhesion formation. Addressing these concerns is essential to improving patient comfort and functional outcomes. This study aimed to assess the immediate effects of vacuum therapy on skin elasticity and pain sensitivity in C-section scar tissue. **Methods**: Thirty-one women with C-section scars older than six months and less than two years participated in an open clinical trial. The skin elasticity was assessed using the Adheremeter, and the pain sensitivity was measured through the Pressure Pain Threshold (PPT) using algometry. The intervention consisted of a 15 min vacuum therapy session using the AeroFlow^®^ device that targeted the scar and surrounding areas. **Results**: The vacuum therapy intervention resulted in significant improvements in the skin elasticity at multiple assessment points, particularly in regions with fascial restrictions (*p* < 0.05). Concurrently, the PPT values decreased, indicating a reduction in pain sensitivity around the scar area (*p* < 0.05). These findings suggest that vacuum therapy may enhance tissue flexibility and alleviate pain in adherent C-section scars. **Conclusions**: This study demonstrated the potential of vacuum therapy as an effective intervention to improve skin elasticity and reduce pain sensitivity in C-section scars. The Adheremeter-based assessment provided valuable insights into the biomechanical properties of scar tissue and supported its use in scar management protocols. This approach offers a promising, non-invasive strategy for personalized scar treatment, ultimately aiming to enhance patient outcomes and quality of life.

## 1. Introduction

Cesarean deliveries (C-sections) are among the most commonly performed surgical procedures for childbirth globally. According to the World Health Organization (WHO), C-sections accounted for around 21% of all births worldwide in 2021, with significant regional differences, particularly showing high rates in Latin America and the Caribbean, where the rates exceed 40% [1,2,3]. While these rates reflect advances in obstetric care, they also contribute to an increased prevalence of post-surgical complications, including scar formation, adhesion development, and impaired tissue elasticity. These complications can lead to significant clinical challenges, including pain, restricted mobility, and difficulties in subsequent surgeries [4,5].

One of the primary concerns with C-sections is the formation of adhesions, which intensify with repeated surgeries, complicating subsequent interventions and elevating postoperative morbidity [6]. Adhesion incidence after a single C-section can reach 24%, rising to as high as 59% in patients with three or more C-sections. These adhesions often occur between the uterine scar and surrounding tissues and are associated with factors such as larger lower-abdominal incisions, maternal age over 35, elevated body mass index (BMI), infections, and surgeries performed during specific periods [7,8,9,10]. These adhesions may lead to complications, including subfertility (3%), chronic pain (6%), intestinal obstruction (0.2%), and difficulties in future abdominal surgeries, underscoring the clinical need for effective scar assessment and intervention strategies.

The accurate assessment of scar characteristics is essential for effective management, as it provides insight into treatment efficacy and can guide therapeutic decisions. In this regard, skin elasticity is a crucial parameter for evaluating scar quality, and various methods were employed to assess it [11,12,13].

The Adheremeter, for example, is a reliable and accessible tool for measuring tissue flexibility, allowing clinicians to monitor scar pliability and resilience post-treatment. Its high reproducibility makes it an effective alternative for evaluating scar elasticity in clinical settings where rapid and repeatable measurements are required [14].

The need for effective interventions in managing C-section scars is becoming increasingly evident due to the functional impairments and discomfort often associated with adhesion formation. Negative pressure therapy, or vacuum therapy, has emerged as a promising approach to address these issues. This technique uses pulsed or continuous negative pressure to stimulate tissue vascularization, promote collagen production, and facilitate cellular debris removal, all of which are essential in wound healing and scar remodeling. Previous studies investigated the benefits of vacuum therapy for burn and traumatic scars, but the literature on its application in C-section scars remains limited [15]. Given its potential to improve scar pliability and reduce pain, further investigation into its efficacy for post-cesarean recovery is warranted.

Understanding how vacuum therapy influences scar elasticity and tissue remodeling is crucial for optimizing post-surgical rehabilitation strategies. Based on these insights, we hypothesized that vacuum therapy will enhance scar tissue elasticity and improve local perfusion, facilitating a more effective healing process.

The objective of this study was to analyze the immediate changes in post-cesarean scar tissue elasticity following a vacuum therapy intervention using the Adheremeter as the primary assessment tool. This approach aimed to provide a deeper understanding of the vacuum therapy’s mechanisms in scar management and to evaluate the clinical applicability of Adheremeter-based elasticity measurements in cesarean section scar treatment.

## 2. Method

### 2.1. Design

This study was an open-label clinical trial with ethical approval from the Human Research Ethics Committee of the University of Málaga, Spain (approval number CEUMA 42-2024-H), reported according to the STROBE declaration and conducted per the Declaration of Helsinki. This study was registered in clinicaltrials.gov with number NCT06057792.

### 2.2. Setting

Patients were recruited at a physiotherapy private clinic in Malaga from September 2023 to December 2023. Information about this study was provided, including information sheets and informed consent from all subjects.

### 2.3. Participants

A total of 31 patients participated in this study. The participants were recruited at the “Clínica Actium” physiotherapy clinic. Once recruited, a physiotherapist conducted the evaluation to determine the participants’ eligibility and inclusion in this study. The participants were thoroughly informed through an information sheet that detailed all the study conditions, and they signed an informed consent form upon meeting the inclusion criteria. The participants had the option to withdraw from this study at any time. This research was conducted in accordance with the Declaration of Helsinki.

### 2.4. Recruitment Procedures

During their first visit to the physiotherapy clinic, all the participants were informed and asked to sign an informed consent form. After obtaining the information and agreeing to participate, all subjects underwent the evaluations.

Throughout the course of the treatment, potential side effects associated with the intervention were documented. The patients participated voluntarily in the project and were already familiar with the procedure, as the physiotherapist in charge is a specialist in this area. Additionally, each step of the treatment was explained in detail during the session. All perceptions or discomfort reported by the patients, as well as any observable signs related to the procedure—such as sensations of discomfort, skin irritation, or other effects—were recorded. During each session, the participants were encouraged to express any concerns or report any issues related to the intervention.

The inclusion criteria were as follows: (i) participants aged 18–60 years; (ii) patients who underwent transversal C-section surgery, with a scar older than six months and less than 2 years; (iii) patients with a post-C-section scar with a fibrotic appearance and possible surrounding fascial restrictions; (iv) patients with a pain score above 5 on the visual analog scale (VAS).

The exclusion criteria were as follows: (i) patients with neurological, inflammatory, or orthopedic injuries that impaired the balance, hearing, vision, or cognitive abilities necessary to answer questions or complete questionnaires; (ii) patients with other types of injuries in the area to be treated; (iii) patients with local problems that could reduce the skin elasticity (e.g., hyperkeratosis); (iv) patients with keloids on the scar.

### 2.5. Description of the Intervention

Each participant underwent two assessments, one before and one after an intervention that involved pulsed and continuous negative pressure. The participants who met the specified inclusion and exclusion criteria outlined above were enrolled in this study.

This study employed vacuum therapy as a novel approach for post-surgical scar rehabilitation. The intervention utilized the AeroFlow^®^ device, a system known for its precision in delivering customizable suction settings. This device’s ability to alternate between pulsed and continuous negative pressure provided a comprehensive approach to addressing scar adhesion and tissue mobility issues, aligning with this study’s therapeutic goals.

The application focused on the target structure (scar), following the protocol established by the manufacturer (INDIBA) to release adhesions between different tissue layers. The treatment involved vacuum therapy via suction cups, which operated automatically at a frequency and intensity that was tolerable for the patient. The treatment protocol was divided into two phases, lasting a total of 15 min. The protocol described by INDIBA incorporates a combination of continuous suction vacuum therapy and pulsed vacuum therapy. This dual approach was designed to optimize the treatment by targeting tissue layers effectively and enhancing the therapeutic outcomes.

This technique is designed to enhance tissue elasticity, reduce adhesions, and improve local blood flow, contributing to better scar rehabilitation and pain management. The AeroFlow^®^ device offers customizable settings, allowing for the configuration of different suction modes—pulsatile or continuous—as well as the ability to adjust the duration of the suction and rest phases for each mode. Additionally, the intensity and total treatment time can be modified, providing a flexible and precise approach tailored to the specific requirements of the intervention.

During the intervention, the patient was positioned in a supine position to ensure comfort and accessibility to the treatment area, allowing the therapist to apply the therapy with precision. The intervention consisted of two different phases:

Phase 1: static cups (5 min). Suction mode: this was a pulsed negative pressure mode, in a square mode, with a power of 70–80 mbar. In the first phase, static plastic cups were used, specifically selecting the smallest available size according to the manufacturer’s specifications. The precise dimensions of these cups were 5 cm × 5 cm. The method involved applying cups according to fascial restrictions. The direction of myofascial restriction was assessed, and two cups were placed. During the first phase of the vacuum therapy treatment, the suction time was 0.4 s, and the rest time was also 0.4 s. After 3–4 pulses per area, the cups were repositioned, following the restriction lines.

Phase 2: dynamic cups (10 min). Suction mode: continuous negative pressure mode with sliding at 60–70 mbar. In this second phase, suction cups with a diameter between 15 mm and 35 mm were used. The cups were slid over the scar, focusing on areas with greater restrictions.

Of the 31 participants enrolled, 30 completed this study, which resulted in an adherence rate of 96.7%. No adverse effects were reported by the participants during or after the intervention, and no observable signs of skin irritation or discomfort were documented. All participants reported feeling comfortable with the procedure, indicating its potential safety and tolerability for use in clinical practice.

### 2.6. Outcomes Measures

#### 2.6.1. Primary Outcome

##### Adheremeter

The Adheremeter is an innovative tool created to assess post-surgical scar adherence by measuring the restriction of scar mobility in four perpendicular directions: cranial, caudal, medial, and lateral. It quantifies how well the scar moves relative to the underlying tissue at its most adherent point when stretched in these directions. The Adheremeter was previously validated in clinical research, where it demonstrated high reliability and reproducibility in measuring tissue mobility and skin elasticity [14]. The device is designed for ease of use, featuring a low-cost, ergonomic structure made of nine concentric rings with radii ranging from 1 mm to 15 mm. These rings are printed on flexible, transparent film, allowing for effective adaptation to different anatomical surfaces.

In use, the Adheremeter is positioned such that its rings align centrally with the scar, keeping the surrounding skin relaxed and nearby joints in a neutral position. The evaluator applies controlled pressure using their thumb near the outer edge of the device, stretching the skin in the four designated directions. Before beginning, the evaluator ensures the patient is comfortable and requests feedback if any discomfort is experienced. The maximum displacement is noted for each direction, and the evaluator ensures the reference point returns to its starting position after the tension is released. If necessary, the measurement is repeated. The entire procedure typically takes only a few minutes per measurement point [14,16].

The results from the four directions are used to compute the Superficial Mobility Index (SMA), providing a reliable, objective measure of the scar’s mobility and flexibility.

The SMA of scar adhesion is a quantitative measure used to assess the mobility of a scar relative to the underlying tissues. This index is calculated using data obtained from an Adheremeter, which measures the resistance to displacement of the scar when force is applied in different directions. The SMA is determined by averaging the displacements in four directions and dividing by the average force applied. A higher SMA indicates greater mobility, while a lower SMA suggests increased adhesion and reduced mobility, potentially requiring therapeutic interventions to improve the elasticity and functionality. This quantitative tool is invaluable for healthcare professionals in scar rehabilitation, as it allows for the objective monitoring of progress and adjustments to therapeutic strategies [14,16,17].

### 2.7. Sample Size

The sample size was determined using G*Power 3.1.9.7 software (Heinrich Heine Universität Düsseldorf, Düsseldorf, Germany) to ensure adequate statistical power. Based on the results of previous studies that evaluated PPT and skin elasticity in patients with nonspecific lumbopelvic pain, a minimum of 18 participants was required to achieve a power of 80% with a significance level (α) of 0.05 and an expected effect size of d = 0.5 (moderate effect size) [18]. To account for an expected dropout rate, the sample size was increased to 31 participants. Ultimately, 30 women completed this study, which provided a robust sample for the preliminary analysis. While this sample size was suitable for detecting moderate changes in skin elasticity and pain sensitivity, larger studies are needed to confirm long-term clinical significance.

### 2.8. Statistical Analysis

All statistical analyses were performed using IBM SPSS Statistics, version 27. Descriptive statistics (mean, standard deviation) were calculated for the demographic variables of age, weight, and height. The normality of the data was assessed using the Shapiro–Wilk test. Given the paired design of this study, a paired *t*-test was used to evaluate the differences in the skin elasticity and Pressure Pain Threshold (PPT) measurements before and after the vacuum therapy intervention. For the non-normally distributed data, the Wilcoxon signed-rank test was applied to assess the differences in the PPT. The effect sizes were calculated using Cohen’s d for the *t*-tests, and rank biserial correlation for the Wilcoxon tests to quantify the magnitude of change. A significance level of *p* < 0.05 was set for all the tests.

## 3. Results

**Figure 1:**
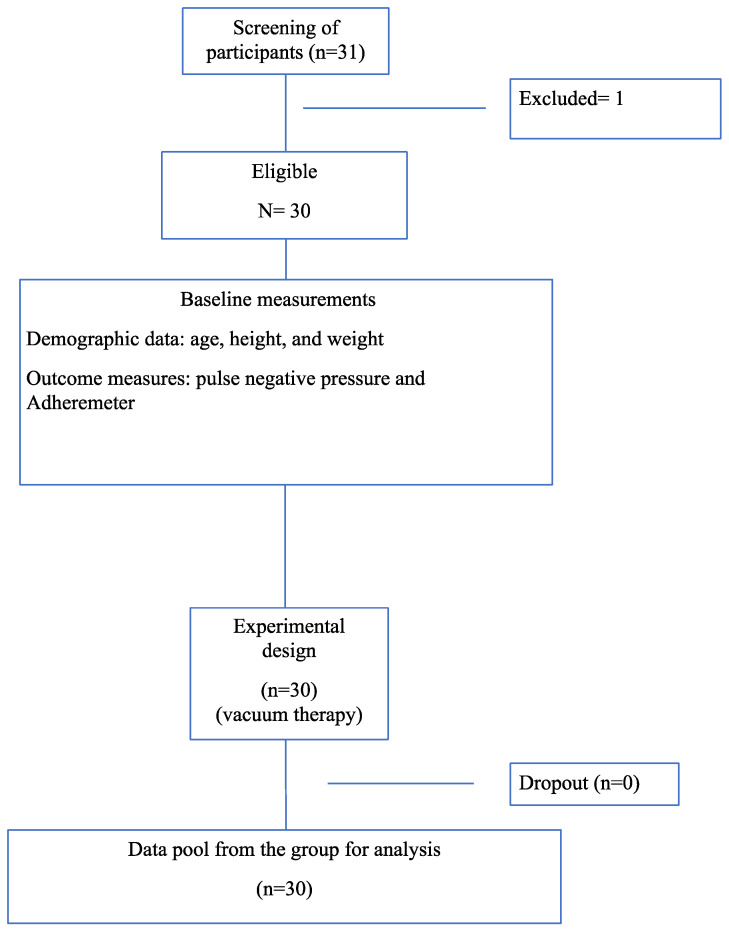
Flow diagram of participants.

A total of 31 participants were initially enrolled in this study; however, the final analysis included 30 participants due to one participant not completing the final evaluation (see Figure 1). The demographic data show that the average age of the participants was 35.9 years (SD = 3.81), with a range from 29 to 46 years. The average weight was 65.7 kg (SD = 11.2), ranging from 48.0 kg to 89.0 kg, and the average height was 1.65 m (SD = 0.0512), ranging from 1.50 m to 1.75 m. The Shapiro–Wilk tests confirmed the normal distributions of age, weight, and height with non-significant *p*-values, indicating that the demographic characteristics were normally distributed across the sample. To provide a more comprehensive understanding of the magnitude of changes observed in the skin elasticity and Pressure Pain Threshold (PPT) results, the effect sizes were calculated using Cohen’s d. This statistical measure quantifies the strength of an effect, allowing for a more meaningful interpretation of the clinical relevance of the findings. Cohen’s d values are commonly categorized as follows: small effect (0.2 ≤ d < 0.5), moderate effect (0.5 ≤ d < 0.8), and large effect (d ≥ 0.8). These classifications provide additional context to the statistical significance of the results, helping to determine the practical implications of vacuum therapy on cesarean scar tissue.

### 3.1. Elastic Changes in Skin After Vacuum Intervention

**Figure 2:**
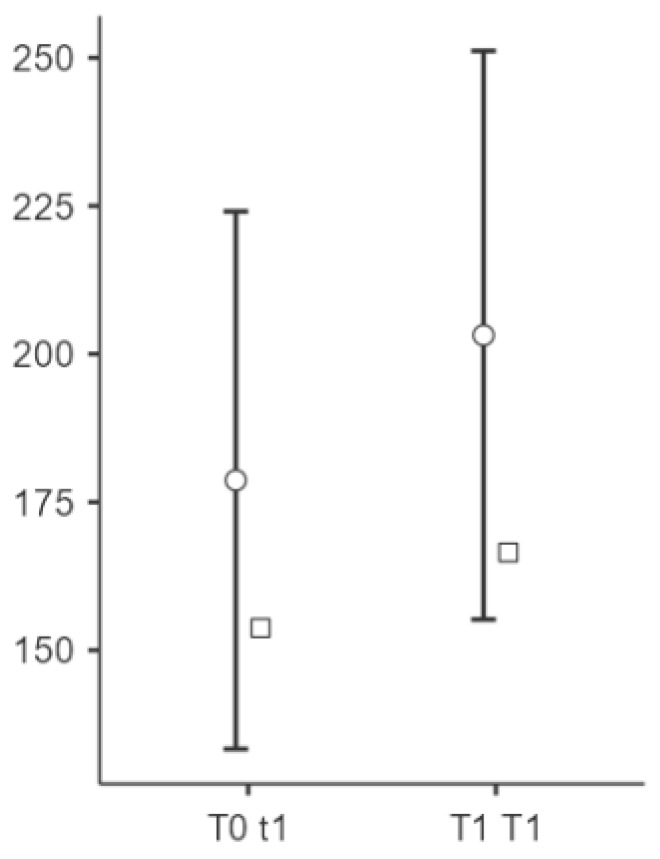
Changes in the skin elasticity at point t1 after the intervention. Notes: The circles represent the mean values with 95% confidence intervals (CIs), while the squares indicate the median values. The variables T (T0) and t (T1) represent the pre-treatment and post-treatment values, respectively. A shift toward more negative values in the *Y*-axis suggests an increase in skin elasticity, indicating improved tissue mobility and flexibility. The left side of the scar (T1) demonstrated a moderate improvement in the elasticity (Cohen’s d = −0.497, *p* = 0.023), meaning that this area showed an intermediate response to the vacuum therapy.

**Figure 3:**
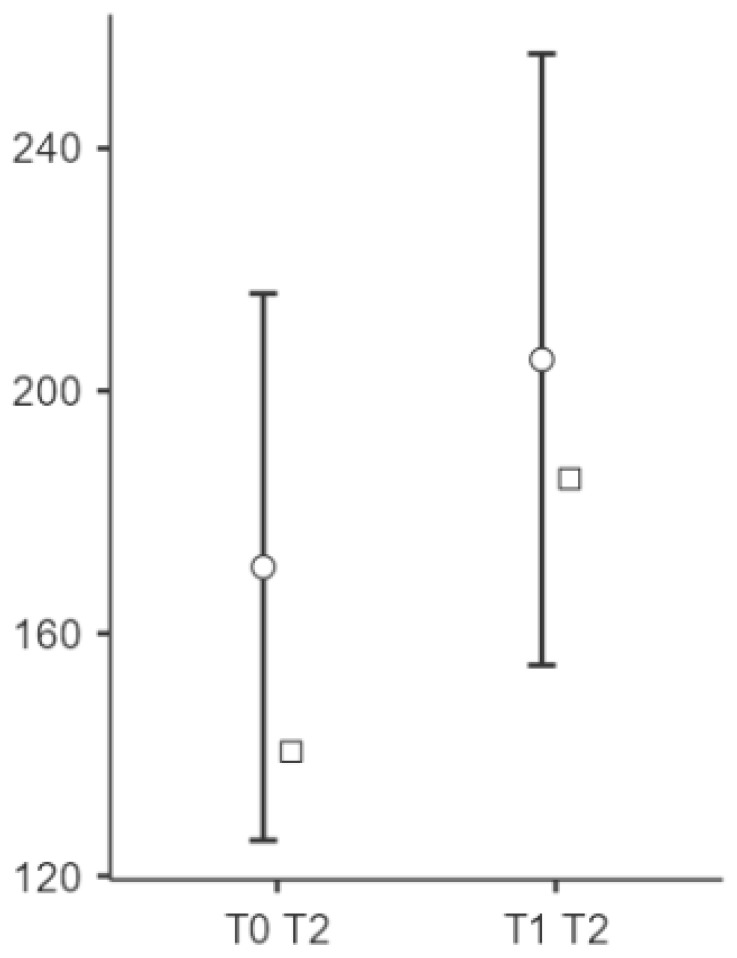
Changes in the skin elasticity at point t2 after the intervention. Notes: The circles represent the mean values with 95% CIs, while the squares indicate the median values. The variables T (T0) and t (T1) correspond to the pre-treatment and post-treatment values, respectively. A greater shift toward negative values on the *Y*-axis at this point (t2) indicates a larger increase in skin elasticity than at T1. The middle of the scar (T2) showed a statistically significant increase in the elasticity (Cohen’s d = −0.643, *p* = 0.004), with a moderate-to-large effect size.

**Figure 4:**
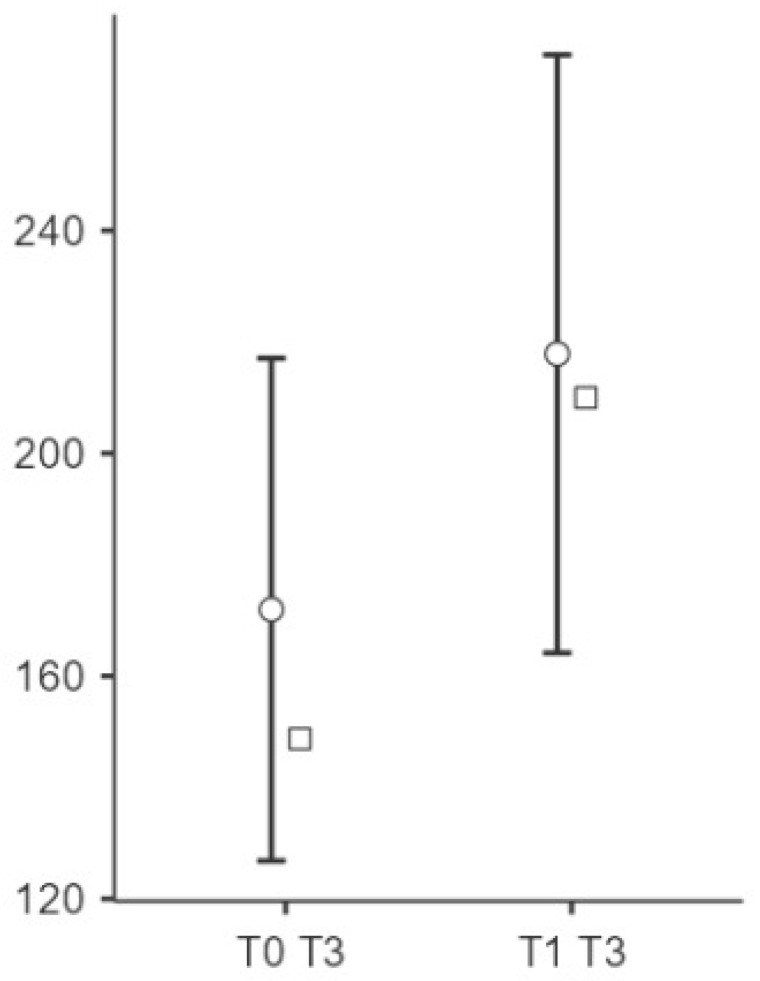
Changes in the skin elasticity at point t3 after the intervention. Notes: The circles represent the mean values with 95% CIs, while the squares indicate the median values. The variables T (T0) and t (T1) correspond to the pre-treatment and post-treatment values, respectively. The most pronounced negative shift in the values on the *Y*-axis was observed at this point (t3), indicating the greatest improvement in the tissue elasticity. The right side of the scar (T3) exhibited the largest increase in the elasticity (Cohen’s d = −0.783, *p* < 0.001), suggesting a strong response to vacuum therapy.

Significant changes were observed in the elasticity of the skin measurements from T0 to T1 at all points (see Figure 2, Figure 3 and Figure 4). Point t1 (left side of the scar) showed a significant improvement in the elasticity (*p* = 0.023), with a mean difference of −24.5 units, 95% CI [−45.3, −3.69], and a medium effect size (Cohen’s d = −0.497). Point t2 (middle point of the scar) showed a further increase (*p* = 0.004), with a mean difference of −34.2 units, 95% CI [−56.7, −11.75], and a medium to large effect size (Cohen’s d = −0.643). Point t3 (right side of the scar) showed significant changes (*p* < 0.001), with a mean difference of −45.9 units, 95% CI [−70.7, −21.13], representing a large effect size (Cohen’s d = −0.783).

### 3.2. PPT Changes After Vacuum Intervention

**Figure 5:**
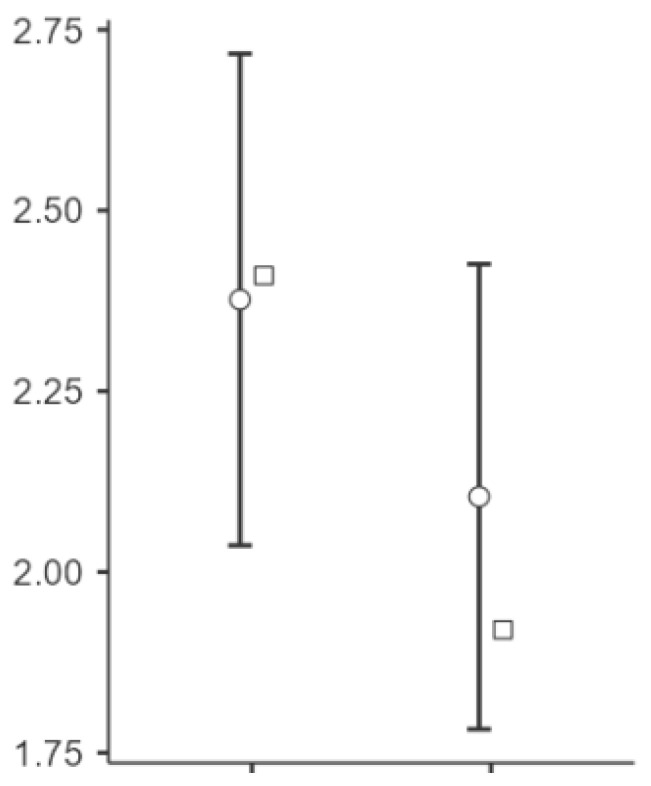
Changes in the PPT at point t1 after the intervention. Notes: Circle means “mean (95% CI)” and square means “median”. Representation of the improvement in the cesarean scar elasticity measured by algometry after an intervention with the AeroFlow^®^ device on the left side of the patient’s scar. A higher PPT value on the *Y*-axis post-intervention (t1) suggests a reduction in pain sensitivity, reflecting an improved tolerance to pressure-induced pain. The left side (T1) showed a statistically significant increase in the PPT (Cohen’s d = 0.333, *p* = 0.018), indicating that the vacuum therapy was effective at reducing the pain perception in this area.

**Figure 6:**
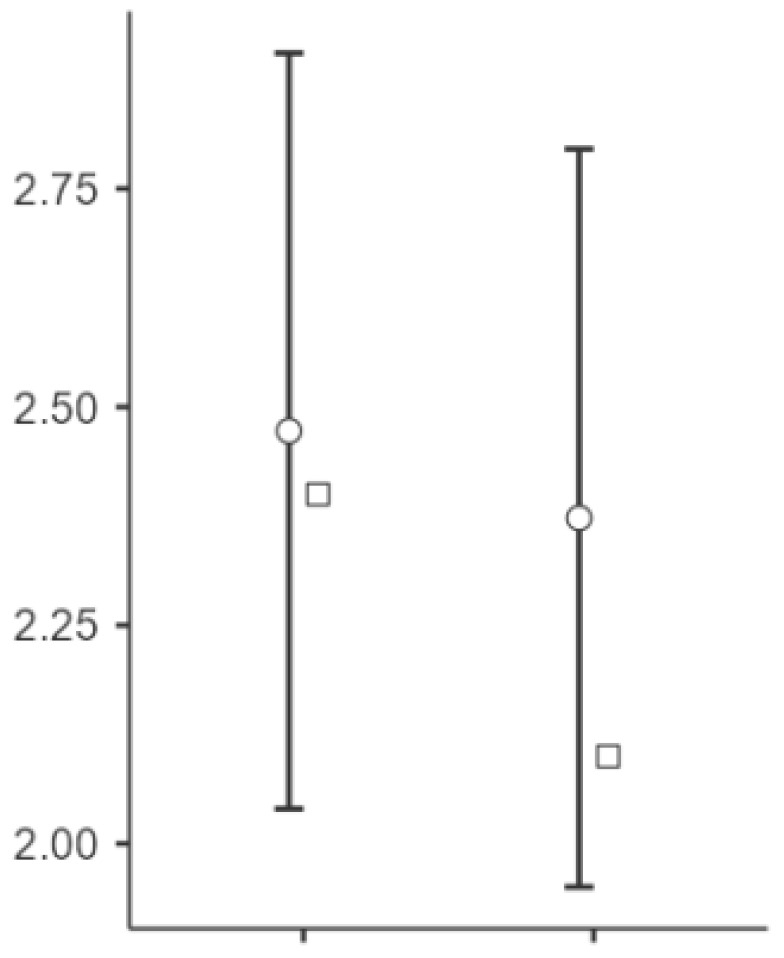
Changes in the PPT at point t2 after the intervention. Notes: Circle means “mean (95% CI)” and square means “median”. The middle region (T2) did not show significant changes in the PPT (Cohen’s d = 0.143, *p* = 0.433), suggesting that vacuum therapy had a limited effect on the pain sensitivity in this area.

**Figure 7:**
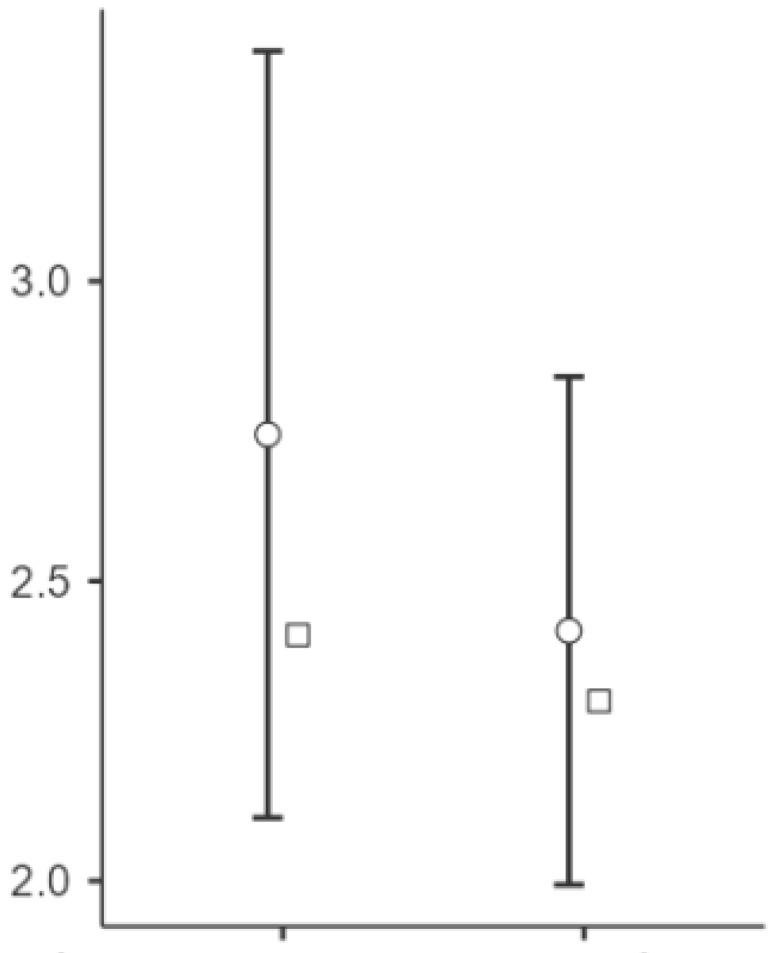
Changes in the PPT at point t3 after the intervention. Notes: Circle means “mean (95% CI)” and square means “median”. The right side (T3) showed a small but non-significant effect on the PPT (Cohen’s d = 0.251, *p* = 0.173). While no statistical significance was observed, a slight increase in the PPT post-treatment (t3) suggests a potential trend toward an improved pain tolerance in this area.

Significant changes were observed in the Pressure Pain Threshold (PPT) measurements from T0 to T1 across all points (see Figure 5, Figure 6 and Figure 7). Point T1 (left side) showed a significant change in the PPT (*p* = 0.018), with a mean difference of 0.310 units, 95% CI [0.075, 0.575], and a small-to-medium effect size (Cohen’s d = 0.333). Point T2 (middle) did not show significant changes (*p* = 0.433), with a mean difference of 0.100 units, 95% CI [−0.156, 0.356], and a small effect size (Cohen’s d = 0.143). Point T3 (right side) also did not reach statistical significance (*p* = 0.173), with a mean difference of 0.327 units, 95% CI [−0.152, 0.807], but indicated a small effect size (Cohen’s d = 0.251).

## 4. Discussion

The aim of this study was to investigate the immediate effects of vacuum therapy on the skin elasticity and Pressure Pain Threshold (PPT) in cesarean section scar tissue. Specifically, we sought to determine whether this intervention could improve these parameters in the scar area. The results demonstrated significant improvements in skin elasticity across all the measurement points, particularly on the left side of the scar, where the most substantial changes were observed. Regarding the PPT, although an improvement trend was evident, statistical significance was only reached on the left side of the scar.

To our knowledge, this was the first study to specifically examine the effects of vacuum therapy on the skin elasticity and PPT in cesarean scar tissue, making direct comparisons with previous research challenging. However, our findings can be contextualized with studies that explored various therapeutic interventions in other types of scars, such as surgical and burn scars. These investigations showed that mechanical stimulation can enhance tissue elasticity, increase fibroblast activity, and facilitate collagen realignment, leading to a reduction in stiffness and pain sensitivity [19,20,21].

Regarding burn scars, for instance, vacuum therapy has been associated with decreased tissue rigidity and discomfort, mainly due to improved circulation and reduced interstitial edema, which lowers the mechanical tension within the scar tissue [19,20,21].

When comparing these findings with our study on cesarean scars, the results closely align. Our study demonstrated that vacuum therapy improved the skin elasticity, particularly on the left side of the scar, and indicated a trend toward reduced pain sensitivity, as evidenced by an increased PPT. The findings of the present study reveal an increase in the PPT after the vacuum therapy was applied to the cesarean scar tissue, suggesting a reduction in the pain sensitivity. An elevated PPT indicates that a higher level of pressure is required before the patient experiences pain, reflecting greater pain tolerance or diminished pain perception [22].

This reduction in sensitivity may be attributed to the positive effects of vacuum therapy on tissue mechanics and circulation, promoting remodeling, reducing adhesions, and improving skin flexibility. These improvements align with previous findings that highlight the role of mechanical stimulation in altering myofascial structure, promoting fibroblast activation, and reorganizing collagen fibers in a more functional manner, which can directly influence pain perception and the mechanical properties of tissue [23,24,25].

Furthermore, increased circulation and lymphatic drainage following vacuum therapy may contribute to a reduction in the inflammatory mediators present in scar tissue, which are known to modulate pain sensitivity [23,24,25]. From a clinical perspective, such improvements imply reduced discomfort associated with scar tissue, likely resulting in better mobility, reduced fascial restrictions, and greater comfort and quality of life for patients during post-operative recovery [26,27].

Scar stiffness has been associated with a reduced range of motion, localized discomfort, and an increased risk of adhesion-related complications, particularly in post-surgical scars. Therefore, the observed increases in elasticity, particularly at T3 (right side of the scar), where the effect size was the largest (Cohen’s d = −0.783, *p* < 0.001), suggest that vacuum therapy may provide a clinically relevant improvement in scar function. Although our study showed statistically significant improvements in elasticity, pain reduction across all points did not reach significance, suggesting potential variations in how different types of scars respond to vacuum therapy.

Accurate assessment is crucial in scar management. The Vancouver Scar Scale (VSS) is the most commonly used tool for evaluating scar characteristics by measuring pigmentation, vascularity, flexibility, and height [28]. However, emerging evidence suggests that combining elastography with the PPT provides additional valuable insights into tissue properties, particularly regarding scar stiffness and its relationship to pain sensitivity. Mechanotransduction, the process by which mechanical forces are converted into cellular biochemical signals, plays a pivotal role, as it affects tissue tensegrity, and thus, influences pain perception. Stiffer areas of the scar, due to altered tensegrity, may transmit mechanical stimuli differently, potentially heightening pain sensitivity in these regions [26,27,28].

Clinically, vacuum therapy provides a customizable, non-invasive treatment option that could be integrated into standard scar management protocols, allowing for personalized adjustments in intensity and frequency based on the patient’s specific needs and stage of recovery. This adaptability, combined with the observed benefits in tissue flexibility and comfort, underscores vacuum therapy’s potential as a supportive intervention in the comprehensive care of post-cesarean patients.

This study presents several strengths that enhance the robustness and relevance of its findings. Primarily, this was the first study to evaluate changes in skin elasticity specifically in cesarean section scars using the Adheremeter, a reliable tool that provides precise, objective measurements of tissue flexibility. By combining these elasticity measurements with PPT assessments, we obtained a comprehensive understanding of the effects of vacuum therapy on both the tissue pliability and pain sensitivity in the scar tissue. Additionally, the homogeneity of the participant group in terms of demographic characteristics reduced the variability and strengthened the internal validity of the results. All measurements were conducted by trained professionals, further ensuring the consistency and data accuracy.

Certain limitations of the present study should be considered. While the sample size was smaller than what might be employed in larger, confirmatory trials, it was suitable for this initial investigation into the immediate effects of vacuum therapy on cesarean section scars. This sample size balanced feasibility, ethical recruitment standards, and this study’s objective to establish foundational insights in this novel area of research, which may serve as a basis for future studies with expanded populations and longitudinal designs. A key limitation of this study was its open-label design, which may introduce biases related to participant and researcher expectations; however, efforts were made to mitigate these biases through the use of objective measurement tools and standardized data collection procedures. Additionally, while the Adheremeter is highly effective in measuring superficial tissue elasticity, factors such as abdominal fat thickness may have influenced the readings [29,30].

The findings of this study suggest that vacuum therapy may offer a promising approach for improving skin elasticity and potentially reducing pain sensitivity in cesarean section scar tissue, with direct implications for clinical practice. However, future studies should establish optimal treatment parameters for vacuum therapy, such as intensity, frequency, and duration, to achieve clinically significant improvements and further explore how increased scar flexibility translates into functional benefits for patients.

Future research should integrate multidisciplinary approaches to better understand the mechanisms underlying scar remodeling. This could include biochemical studies that focus on molecular pathways, such as the role of TGF-β1 and the TGF-β1/Smad signaling cascade in collagen deposition and myofibroblast contraction [19,31,32,33], which significantly contribute to scar stiffness and hypertrophy. Vacuum therapy may influence these pathways through mechanotransduction, facilitating fibroblast reorientation and collagen realignment [19,33]. Additionally, recent studies suggested that mechanical stimulation may play a role in modulating inflammatory mediators, including cytokines, such as IL-6 and TNF-α, which are involved in chronic pain pathways. This could further support the hypothesis that vacuum therapy contributes to pain modulation not only through mechanical effects but also through biochemical regulation.

The investigation of markers such as α-SMA could provide deeper insights into tissue stiffness and the physiological impact of therapeutic interventions. Histological analyses of patients undergoing repeated cesarean sections or similar procedures could offer a valuable perspective on scar tissue properties and help validate clinical findings. Experimental models using human-derived skin grafts in animals have confirmed that therapeutic strategies targeting early scar remodeling can mitigate hypertrophic changes and improve tissue elasticity [33]. Incorporating these data into future studies will enable a more comprehensive understanding of the underlying mechanisms and expand the clinical implications of vacuum therapy.

The application of elastography as an assessment tool could further enhance future research on scar tissue properties. Elastography allows for the non-invasive, precise measurement of tissue stiffness and elasticity, providing valuable insights into the structural and mechanical changes that occur in scar tissue during and after treatment.

Studies demonstrated that cesarean incision scars exhibit significantly higher stiffness compared with surrounding tissues, likely due to increased collagen content and altered biomechanical properties resulting from the healing process [6,34]. Additionally, research suggests that the presence of intra-abdominal adhesions in repeated cesarean sections could also influence the biomechanical properties of the scar, leading to changes in the mechanical stress distribution and pain perception [10,35]. This highlights the need for an integrative approach that considers both superficial and deep tissue changes in post-cesarean recovery.

For instance, Di Pasquo et al. quantified uterine scar stiffness and observed that elastography reliably distinguished scar tissue from intact myometrium, suggesting its utility in assessing post-surgical outcomes and predicting complications, such as a uterine rupture, in future pregnancies [34]. Similarly, Seven et al. highlighted a correlation between subcutaneous tissue stiffness and intra-abdominal adhesions, emphasizing the potential of shear wave elastography (SWE) to inform surgical planning and reduce intraoperative complications in repeated cesarean deliveries [6]. The incorporation of this technique into future studies would not only provide more objective data on tissue flexibility but also improve the ability to monitor therapeutic progress, enhance personalized treatment strategies, and standardize long-term outcome assessments. The combination of elastography with clinical and biochemical analyses could provide a more comprehensive understanding of the effects of vacuum therapy on scar tissue and improve real-time evaluations and personalized treatment planning.

Additionally, future studies should consider differentiating patients based on demographic characteristics, particularly in relation to the abdominal fat percentage and body weight, as these factors may influence the mechanical properties of scar tissue and its response to vacuum therapy. Understanding how variations in body composition affect treatment efficacy could lead to more personalized therapeutic approaches, optimizing intervention strategies for different patient profiles. For example, higher abdominal fat levels may alter the effectiveness of vacuum therapy due to differences in tissue compression and local circulation, requiring modified treatment intensities or durations for optimal outcomes. Similarly, variations in BMI could influence PPT measurements and tissue elasticity, warranting further stratification in future analyses.

## 5. Conclusions

This exploratory study demonstrated that vacuum therapy appears to have a significant effect on skin elasticity and a moderate effect on the Pressure Pain Threshold in cesarean section scar tissue. These preliminary findings indicate that vacuum therapy could be a promising intervention for improving tissue flexibility and reducing pain sensitivity in post-surgical scars, which may enhance patient comfort and quality of life. However, the results should be interpreted with caution, as further research is necessary to confirm these findings, investigate underlying mechanisms, and assess the long-term effects and broader applicability of this intervention.

## Data Availability

All data associated with this study are present in this paper. All requests for other materials will be reviewed by the corresponding author to verify whether the request is subject to any intellectual property or confidentiality obligations.
